# Bone stimulation for fracture healing: What's all the fuss?

**DOI:** 10.4103/0019-5413.50844

**Published:** 2009

**Authors:** Galkowski Victoria, Brad Petrisor, Brian Drew, David Dick

**Affiliations:** Center for Bone Healing and Research, Performance Physiotherapy, Hamilton, Ontario, Canada

**Keywords:** Electrical stimulation, electrical stimulation therapy, fracture healing, low-intensity pulsed ultrasound, pulsed electromagnetic fields

## Abstract

Approximately 10% of the 7.9 million annual fracture patients in the United States experience nonunion and/or delayed unions, which have a substantial economic and quality of life impact. A variety of devices are being marketed under the name of “bone growth stimulators.” This article provides an overview of electrical and electromagnetic stimulation, ultrasound, and extracorporeal shock waves. More research is needed for knowledge of appropriate device configurations, advancement in the field, and encouragement in the initiation of new trials, particularly large multicenter trials and randomized control trials that have standardized device and protocol methods.

## INTRODUCTION

The economic and health burden of fractures is large. Fortunately, most fractures heal without any complications. However, out of the estimated 7.9 million fractures that occur annually in the United States, 5–10% of them develop nonunions and/or delayed unions, which are major sources of complications in the treatment of bone fractures.^1^ Fracture healing is a complicated metabolic process and requires the interaction of many factors, including the recruitment of reparative cells and genes. If these factors are inadequate or interrupted, healing is delayed or impaired, resulting in a nonunion of the bone.^2^

The cause of nonunions and delayed healings of fractures is usually unknown. The known reasons of delayed or impaired unions include problems with operative and nonoperative interventions, comprising inadequate mobilization of the fracture, distraction of fracture fragments by fixation devices or traction, repeated manipulations or excessive early motion of a fracture, excessive periosteal stripping, and damage to other soft tissues during operative exposure. Other risks for impaired fracture healing include contamination at the time of injury or operation, smoking, diabetes, and the skeletal location of the injury.^3^

Bone healing may be manipulated by external (biomechanical) and internal (biological) stimuli. The ability for fracture healing to be enhanced in the percentage of patients with impaired fracture healing would have a great economic impact, as well as enhance the physical and mental well-being of these patients. A variety of biological, mechanical, and physical interventions have been developed to enhance fracture healing. This article focuses on the range of physical methods to stimulate bone healing including electrical stimulators, low-intensity pulsed ultrasound, and extracorporeal shock waves. These modalities are less invasive to patients and the cost or complications related to harvesting an autograft are eliminated.^3^

## HISTORICAL PERSPECTIVES

There have been case reports of success using electrical stimulation as early as 1841,^4^ but the use of this method of treatment did not progress until the 1950s. In 1953, Yasuda applied continuous current to a rabbit femur for three weeks and demonstrated new-bone formation in the vicinity of the cathode.^5^ It became known that there are electrical potentials in bone, including stress-generated potentials^5^ and bioelectric or steady-state potentials.^3^ Stress-generated potentials occur when a portion of bone is subjected to a bending load and that portion becomes electronegative, while other tensile parts become electropositive. Bioelectric potentials are electronegative potentials that occur in nonstressed bone in areas of active growth and repair. Investigators around the world began to study the effects of electricity on bone and cartilage, and by 1976, at least 119 articles appeared in the world literature describing the effects of different forms of electricity on bone growth and repair.^3^ A variety of electrical stimulation devices have now been developed.

Another physical stimulus that is of newer use in the enhancement of bone healing is sound. The benefits of ultrasound are determined by intensity. Diagnostic use of ultrasound requires very low intensities (milliwatts per square centimeter) to avoid excessive heating of the tissues. Nevertheless, ultrasonic intensities of one to three watts per square centimeter have been reported to reduce joint stiffness, pain, muscle spasm, improve muscular mobility, and more recently enhance the growth and healing of bones.^3^ A report of low-intensity ultrasounds playing a role in bone growth and fresh fracture healing of rabbits was published in 1983^6^, and the first clinical application of ultrasounds on the treatment of nonunions was read at the Annual Meeting of The American Academy of Orthopaedic Surgeons in 1987 by Duarte and Xavier.^3^ Throughout the subsequent years, low-intensity pulsed ultrasounds have been shown to be effective in the treatment of upper and lower extremity fractures. Thus, in 1994, the Food and Drug Administration (FDA) approved the marketing of ultrasounds for the healing of fresh fractures.^7^

An even more recent method, now being studied for the treatment of bone fractures, is extracorporeal shock waves (ESWT). This method requires higher frequencies and energies and has been used as a standard for the treatment of ureter stones. In recent years, investigators have become interested in ESWT absorption through bone structures. Only a few studies have been published so far on the mechanisms and effectiveness of this therapy.^8^

At this time, the various bone stimulation devices are being produced and marketed—under the common names, “external bone growth stimulators” and “implantable bone growth stimulators.” Some of the common companies include Biomet Incorporated, Smith and Nephew Incorporated, DJO Incorporated, Depuy Spine, Orthofix Incorporated, and VQ OrthoCare.

## BONE STIMULATORS: HOW DO THEY WORK?

### Electrical stimulation

Electrical and electromagnetic (EM) fields are assumed to play a role in bone healing through the same principles as mechanical stress applications. When mechanical load is applied to bone, a strain gradient develops.^4^ Subsequent pressure gradients in the interstitial fluid drive fluid through the canaliculi from regions of high to low pressure and expose osteocyte membranes to flow-related shear stress, as well as to electrical potentials subsequent to the streaming process.^4^ Application of EM to the fracture site is meant to mimic the effect of mechanical stress on bone.

A variety of instruments have been developed to be delivered to electrical and EM fields to fracture sites, each being categorized into one of three types: invasive direct-current (DC) stimulators, noninvasive capacitive coupling (CC) stimulators, and noninvasive inductive coupling (IC) stimulators—produced by pulsed electromagnetic fields (PEMF).

However, the effects of EM on cellular processes are not well understood.^4^ Aaron *et al.*,^9^ reviewed a series of preclinical and clinical studies on electrical and electromagnetic energy on bones. Applications of PEMF and their role on regulation of structural ECM proteins have been explored in more details than the other two electrical stimulation techniques. Preclinical studies, both *in vitro* and *in vivo*, have demonstrated that EM stimulates the synthesis of structural extracellular matrix (ECM) proteins and initiates cascade events in the production of proteins that have a role in gene regulation and signal transduction of electrical potentials.^10^ Many studies have observed the upregulation of mRNA levels and protein synthesis for growth factor, which enhances cellular repair and the synthesis of ECM proteins.^4^ It has been demonstrated that the amplification of the electrical and electromagnetic fields are probably due to transmembrane receptors (including PTH, insulin, IL-2, transferrin, LDL, IGF-2, calcitonin, and adenosine A_2A_).^10^ Electrical stimulators have also been used and studied clinically, specifically, for their efficacy in fresh fractures and osteotomies, spine fusions, and delayed and nonunion of fractures. There is no standard on configuration and dose of electric or electromagnetic input, and these specific settings may determine which transmembrane signaling mechanisms are activated.^10^

Direct-current stimulators deliver EM though either implanted or percutaneously applied insulated electrodes.^11^ In surgically implanted electrodes, the cathode is placed into the site of bone repair, while the anode is placed in nearby soft tissues. The power sources and generating units can be external or implanted. The current is applied constantly by the power generators for several months, and osteogenesis is stimulated at the cathode at currents of 5–100 μA.^9^ In DC stimulation, a dose-response curve has been shown where currents below a certain threshold lead to bone formation, while those above a certain threshold show cellular necrosis.^3^

Stimulation via CC devices usually applies potentials of 1–10 V at frequencies of 20–200 kHz. The resulting electrical fields in the tissue are around 1–100 mV/cm. These devices are noninvasive and the electrodes are placed on the skin on opposite sides of the fracture site.^9^

The third technique that has become quite popular is IC stimulation, which is also applied externally (as the CC technique), and it produces electrical fields in bone with varying or pulsed electromagnetic fields (hence this technique is also referred to as PEMF).^9^ The current is produced by a single or double coil, driven by an external field generator. The outcome is a secondary electrical field produced in the bone. Both the characteristics of the applied magnetic fields and the biological properties of the tissues influence the induced secondary field. In practice, the configurations of the applied magnetic fields have varied by amplitude, frequency—single pulse or pulse burst (a serious of pulses with frequencies of 1 to 100 bursts/second)—and wave form. Varying configurations have produced magnetic fields of 0.1–20 G, which have produced voltage gradients of 1–100 mV/cm.^9^

The advantages of electrical stimulation may be the low complication rates as compared to other invasive methods. Implantable forms of the DC stimulators have the advantage of providing constant stimulation of bone directly at the fracture site as well as increased patient compliance. However, the invasive DC method may cause more infection rates, have the potential for a painful implant, and the common stress associated with operative procedures.^11^ There is a great need for thorough explorations of success rates and cost-effectiveness of electrical stimulation methods compared to performing another surgery on patients with nonunion or malunion).

### Low-intensity pulsed ultrasound

*In vitro* studies suggest that ultrasonic stimulation enhances bone healing by increasing the incorporation of calcium ions in cultures of cartilage and bone cells and stimulate the expression of numerous genes (including genes for Aggrecan, IGF, and TGF-β) involved in the healing process.^4^ The most important effect that ultrasound has on bone healing may be on chondrocyte population, as suggested by studies that demonstrate an increase in the formation of soft callus and early onset of endochondral ossification after ultrasonic applications.^4^ Many preclinical and clinical studies have demonstrated promising results using low-intensity pulsed ultrasounds for healing fresh fractures and treatment of delayed union or nonunions.

The ultrasonic intensity required to heal fractures is lower (not exceeding 30 W/cm^2^) than that currently used by physiotherapists (spatial-averaged temporal-averaged intensities ranging from 2 to 100 W/cm^2^). Although ultrasound has been used for healing purposes, many textbooks, including reviews on fracture management, but specifically occupational therapy and physiotherapy texts, continue to “misclassify” the use of ultrasound for the treatment of fractures as a contraindication. These notions are largely based on much higher intensity ultrasound (100 W/cm^2^) using the physiotherapy literature; damage to tissues has been demonstrated by the use of high intensity ultrasonography.^12^

### Extracorporeal shock waves

Extracorporeal shock waves (ESWT) have very recently started being investigated, and the mechanisms of action are not well known or researched. The therapy is not currently used as a standard treatment for bone fractures.^8^

## CURRENT EVIDENCE FOR BONE STIMULATORS

The effect of electrical stimulators on the enhancement of fresh fracture healing remains inconclusive. Researchers have had mixed results in answering whether the use of electrical stimulators enhances the healing of slow-to-heal fractures.^11^ Most of the studies, however, have not been of high methodological quality. Meta-analyses on the efficacy of electrical stimulators on bone repair have been difficult to perform because of the heterogeneity of study designs and outcome measurements and inability to pool the data of various studies.

A recent meta-analysis of electrical stimulation for long-bone fractures^13^ identified 11 studies (of variable methods, device administration, and quality) for analysis. Although conclusions were limited, the authors reported that electromagnetic stimulation resulted in a short-term increase in scintimetric healing activity on in nonoperatively treated Colles fractures, bone density is improved in patients undergoing femoral intertrochanteric osteotomy, and bone density is variably impacted in lengthening procedures of the lower limb.

Low-intensity pulsed ultrasound, on the other hand, has a fairly extensive evidence base derived from randomized trials. In particular, one meta-analysis of 3 studies was conducted to explore the effect of low-intensity pulsed ultrasound therapy on time to fracture healing.^12^ The studies that were pooled had one group of patients receiving low-intensity ultrasound treatment and one control group in examining the treatment of scaphoid, distal radial, and tibial shaft fractures. The pooled results for the studies showed that the time of healing in the ultrasound group was significantly shorter than in the control group (the weighed average effect size being 6.41 with 95% confidence interval of 1.01–11.81); the mean difference in healing time was calculated to be 64 days. These findings suggest that ultrasound may have substantial benefits to both quality of life and cost effectiveness in fracture healing.

## Current Trends in Bone Stimulation Use

The most commonly used bone stimulators are the low-intensity pulsed ultrasounds and electrical stimulation devices. Frost and Sullivan market research specialists report that sales of these devices, especially noninvasive spinal fusion stimulators, are climbing.^14^ Pulse electromagnetic field (PEMF) stimulators are the most commonly used type of noninvasive bone growth and spinal fusion stimulators.

In North America, there is a rather wide use of bone stimulation therapies for tibial shaft fractures, the most common of all long-bone fractures. Busse *et al.*,^15^ conducted a survey to explore current management of tibial shaft fractures among Canadian orthopedic surgeons. Most survey respondents had been in practice for more than 10 years, managing mostly closed tibial shaft fractures, and results are limited to generalization to surgeons within the Canadian Orthopaedic Association. Most respondents (80%) considered a reduction in tibial shaft fracture healing time of 6 weeks to be a clinically important reduction. Although evidence for effectiveness of these therapies is mixed, almost half of the respondents currently make use of bone stimulators as part of their management of complicated closed fractures and complicated open fractures (45 and 43% of respondents, respectively)–“complicated” being defined as displaying nonunion, delayed union, or malunion. These orthopedic surgeons had an equal preference for electrical stimulators and low-intensity pulsed ultrasound. 3% favored “other” bone stimulators. Based on this survey, Mollon *et al.*,^13^ argued that the current evidence on the effectiveness of electromagnetic stimulation does not support its rather high clinical use among this sample of Canadian orthopedic surgeons. However, the authors did mention that there is a lot of heterogeneity in studies, and more quality studies need to be conducted for stronger meta-analyses and conclusions to be made on the use of electromagnetic stimulation therapies.

Busse and Bhandari^16^ administered a smaller survey of beliefs and practices, regarding the use of ultrasound for bone healing, among orthopedic surgeons, senior physiotherapy (PT) students, and senior orthopedic surgery residents at a Canadian University. Ultrasound use among this group was rare, and many clinicians perceived that there is a lack of evidence and availability for its use, in addition to the belief that ultrasound is contraindicated for the treatment of fractures (consistent with some research and most PT texts).

## DO WE HAVE ENOUGH DATA ON BONE STIMULATORS?

Although multiple randomized trials exist to support the variety of bone stimulation modalities, all are small and limited to primarily radiologic endpoints. There remains a need to conduct, large, and definitive trials that use patient-important outcomes before widespread (and universal) acceptance of such modalities will occur.

## References

[1] (2000). Musculoskeletal injuries report: Incidence, risk factors and prevention.

[2] Childs SG (2003). Stimulators of bone healing: Biologic and biomechanical. Orthop Nurs.

[3] Einhorn TA (1995). Enhancement of fracture-healing. J Bone Joint Surg Am.

[4] Hannouche D, Petite H, Sedel L (2001). Current trends in the enhancement of fracture healing. J Bone Joint Surg Br.

[5] (1953). The classic: Fundamental aspects of fracture treatment by Iwao Yasuda, reprinted from J Kyoto Med Soc.

[6] Duarte LR (1983). The stimulation of bone growth by ultrasound. Arch Orthop Trauma Surg.

[7] (1994). Food and Drug Administration: Talk Paper. Rockville, Maryland. Food and Drug Administration. United States Department of Health and Human Services.

[8] Birnbaum K, Wirtz DC, Siebert CH, Heller KD (2002). Use of extracorporeal shock-wave therapy (ESWT) in the treatment of non-unions. A review of the literature. Arch Orthop Trauma Surg.

[9] Aaron RK, Ciombor DM, Simon BJ (2004). Treatment of nonunions with electric and electromagnetic fields. Clin Orthop Relat Res.

[10] Ciombor DM, Aaron RK (2005). The role of electrical stimulation in bone repair. Foot Ankle Clin.

[11] Haddad JB, Obolensky AG, Shinnick P (2007). The biologic effects and the therapeutic mechanism of action of electric and electromagnetic field stimulation on bone and cartilage: New findings and a review of earlier work. J Altern Complement Med.

[12] Mollon B, da Silva V, Busse JW Einhorn TA, Bhandari M (2008). Electrical stimulation for long-bone fracture-healing: A meta-analysis of randomized controlled trials. J Bone Joint Surg Am.

[13] Frost and Sullivan Research U.S. Bone Growth and Spinal Fusion Stimulators Markets.

[14] Busse JW, Morton E, Lacchetti C, Guyatt GH, Bhandari M (2008). Current management of tibial shaft fractures: A survey of 450 Canadian orthopedic trauma surgeons. Acta Orthop.

[15] Busse JW, Bhandari M (2004). Therapeutic ultrasound and fracture healing: A survey of beliefs and practices. Arch Phys Med Rehabil.

[16] Busse JW, Bhandari M, Kulkarni AV, Tunks E (2002). The effect of low-intensity pulsed ultrasound therapy on time to fracture healing: A meta-analysis. CMAJ.

